# Global decomposition experiment shows soil animal impacts on decomposition are climate-dependent

**DOI:** 10.1111/j.1365-2486.2008.01672.x

**Published:** 2008-11

**Authors:** DIANA H WALL, MARK A BRADFORD, MARK G ST JOHN, JOHN A TROFYMOW, VALERIE BEHAN-PELLETIER, DAVID E BIGNELL, J MARK DANGERFIELD, WILLIAM J PARTON, JOSEF RUSEK, WINFRIED VOIGT, VOLKMAR WOLTERS, HOLLEY ZADEH GARDEL, FRED O AYUKE, RICHARD BASHFORD, OLGA I BELJAKOVA, PATRICK J BOHLEN, ALAIN BRAUMAN, STEPHEN FLEMMING, JOH R HENSCHEL, DAN L JOHNSON, T HEFIN JONES, MARCELA KOVAROVA, J MARTY KRANABETTER, LES KUTNY, KUO-CHUAN LIN, MOHAMED MARYATI, DOMINIQUE MASSE, ANDREI POKARZHEVSKII, HOMATHEVI RAHMAN, MILLOR G SABARÁ, JOERG-ALFRED SALAMON, MICHAEL J SWIFT, AMANDA VARELA, HERALDO L VASCONCELOS, DON WHITE, XIAOMING ZOU

**Affiliations:** * Natural Resource Ecology Laboratory and Department of Biology, Colorado State UniversityFort Collins, CO 80523, USA; † Odum School of Ecology, University of GeorgiaAthens, GA 30602, USA; ‡ Landcare ResearchPO Box 40, Lincoln 7640, New Zealand; § Canadian Forest Service, Pacific Forestry CentreVictoria, BC, Canada V8Z 1M5; ¶ Agriculture and Agri-food CanadaOttawa, ON, Canada K1A 0C6; ∥ Queen Mary University of LondonLondon E1 4NS, UK; ** Macquarie UniversitySydney, NSW 2109, Australia; †† Natural Resource Ecology Laboratory, Colorado State UniversityFort Collins, CO 80523, USA; ‡‡ Institute of Soil Biology, Academy of SciencesČeské Budějovice 370 05, Czech Republic; §§ Institute of Ecology, University of JenaJena 07743, Germany; ¶¶ Department of Animal Ecology, Justus-Liebig-UniversityD-35392 Giessen, Germany; ∥∥ Kenya Methodist University, Kaaga CampusMeru, Kenya; *** Forest Entomology, Forestry TasmaniaHobart, TAS 7000, Australia; ††† Centralno-Chernozemnyj ReserveZapovednoe, Russian Federation; ‡‡‡ MacArthur Agro-Ecology Research CenterLake Placid, FL 33852, USA; §§§ Laboratoire MOST Centre IRD, Institut de Recherche pour le DéveloppementUR SeqBio, SupAgro, Montpellier, France; ¶¶¶ Gros Morne National Park, Rocky HarbourNL, Canada A0K 4N0; ∥∥∥ Gobabeb Training & Research CentreBox 953, Walvis Bay, Namibia; **** Department of Geography, University of LethbridgeLethbridge, AB, Canada T1K 3M4; †††† Cardiff School of Biosciences, Cardiff UniversityCardiff CF10 3US, UK; ‡‡‡‡ Institute of Botany, Academy of SciencesPruhonice 252 43, Czech Republic; §§§§ B.C. Ministry of ForestsSmithers, BC, Canada V0J 2N0; ¶¶¶¶ Inuvik Research CentreInuvik, NT, Canada X0E 0T0; ∥∥∥∥ Taiwan Forestry Research InstituteTaipei 100, Taiwan; ***** Institute of Tropical Biology and Conservation, Universiti Malaysia SabahSabah, Malaysia; ††††† Institut de Recherche pour le DéveloppementOuagadougou 01 BP182, Burkina Faso; ‡‡‡‡‡ Institute of Ecology and Evolution of RASMoscow 119071, Russian Federation; §§§§§ Centro Universitário do Leste de Minas GeraisCoronel Fabriciano 35170-056, Brazil; ¶¶¶¶¶ Tropical Soil Biology & Fertility Institute of CIATICRAF, Nairobi, Kenya; ∥∥∥∥∥ Pontificia Universidad JaverianaBogotá, DC, Colombia; ****** Institute of Biology, Federal University of UberlândiaCP 593, 38400-902 Uberlândia, Brazil; †††††† Forest Resources, Department of Indian and Northern AffairsWhitehorse, YT, Canada Y1A 2B5; ‡‡‡‡‡‡ Xishuangbanna Tropical Botanical Garden, The Chinese Academy of SciencesKunming, Yunnan 650223, China; §§§§§§ Institute for Tropical Ecosystem Studies, University of Puerto RicoSan Juan 00931-1910, Puerto Rico

**Keywords:** climate decomposition index, decomposition, litter, mesofauna, soil biodiversity, soil carbon, soil fauna

## Abstract

Climate and litter quality are primary drivers of terrestrial decomposition and, based on evidence from multisite experiments at regional and global scales, are universally factored into global decomposition models. In contrast, soil animals are considered key regulators of decomposition at local scales but their role at larger scales is unresolved. Soil animals are consequently excluded from global models of organic mineralization processes. Incomplete assessment of the roles of soil animals stems from the difficulties of manipulating invertebrate animals experimentally across large geographic gradients. This is compounded by deficient or inconsistent taxonomy. We report a global decomposition experiment to assess the importance of soil animals in C mineralization, in which a common grass litter substrate was exposed to natural decomposition in either control or reduced animal treatments across 30 sites distributed from 43°S to 68°N on six continents. Animals in the mesofaunal size range were recovered from the litter by Tullgren extraction and identified to common specifications, mostly at the ordinal level. The design of the trials enabled faunal contribution to be evaluated against abiotic parameters between sites. Soil animals increase decomposition rates in temperate and wet tropical climates, but have neutral effects where temperature or moisture constrain biological activity. Our findings highlight that faunal influences on decomposition are dependent on prevailing climatic conditions. We conclude that (1) inclusion of soil animals will improve the predictive capabilities of region- or biome-scale decomposition models, (2) soil animal influences on decomposition are important at the regional scale when attempting to predict global change scenarios, and (3) the statistical relationship between decomposition rates and climate, at the global scale, is robust against changes in soil faunal abundance and diversity.

## Introduction

The annual global release of carbon to the atmosphere through decomposition of organic carbon by soil biota is approximately 50–75 Pg, nearly 10 times that of fossil fuel emissions (Schimel *et al.*, 1996). Climate and plant litter quality (i.e. chemical composition) are considered the primary regulators of litter decomposition, explaining as much as 65–77% of the variation in decomposition rates (Moorhead *et al.*, 1999; Gholz *et al.*, 2000; Trofymow *et al.*, 2002;). The residual variation (ca. 25%) in global decomposition rate models remains a substantial source of error in estimates of contemporary and future global carbon dynamics (Schimel *et al.*, 1996; Del Grosso *et al.*, 2005;).

Putatively, the unallocated error in global decomposition models has a significant biological component, for example organismal biomass, size distribution, taxonomic richness, and/or functional group composition. These components are normally considered as either the direct effects of physical conditions (through addition or removal of habitats and therefore species) or indirect effects (delivered via multiple climate effects defining plant communities and the area of distribution available to major groups of soil organisms). Consequently, biota (animals and microbes) are not explicitly considered in global decomposition models (VEMAP, 1995; Moorhead *et al.*, 1999; Gholz *et al.*, 2000; Lavelle *et al.*, 2004;), although conceptually considered to be key drivers of litter decomposition rates (e.g. Wardle, 1995; Lavelle, 1997; Coleman & Hendrix, 2000; Kibblewhite *et al.*, 2008;). Instead, soil biota have been evaluated (relative to nonbiotic agencies) for their contribution to aggregated ecosystem functions (or services, sensu Daily *et al.*, 1997; Wall, 2004;), including decomposition, together with the related processes of carbon sequestration and greenhouse gas emission. To refine this concept, ecosystem services have been apportioned to functional assemblages of named organisms (for example Lavelle, 1997; Swift *et al.*, 2004; Kibblewhite *et al.*, 2008;), such that the effects of specific disturbances on the delivery of individual services can be elucidated (Wardle, 2002). The modeling, however, is at best semiquantitative and, most crucially, it is ecosystem-specific and cannot be deployed much beyond the landscape scale or made responsive to the incremental changes in physical environmental parameters inherent in climate change scenarios. To factor soil biota into future global decomposition modeling, it is necessary to assess decomposition against a biotic assemblage character on a supraregional scale and by an experimental procedure that will adequately distinguish between the abiotic and the biotic agencies responsible for the process.

Almost 30 years ago, Swift *et al*. (1979) hypothesized that the relative contribution of soil fauna (vs. microflora) to decomposition was dependent on the climatic region, being greatest at midlatitudes and decreasing towards the poles. In contrast to the many multisite experiments on climate and litter quality (Moorhead *et al.*, 1999; Trofymow *et al.*, 2002;), relationships between soil animals and litter decomposition have never been experimentally tested at global or regional scales. Furthermore, there are few data to judge the significance of changes in diversity of soil fauna at these scales (Swift *et al.*, 1998), which reflects the scarcity of the required taxonomic expertize and the prohibitive effort needed to characterize biota in a sufficient number of sites and with a resolution that reflects true biodiversity. Inferences from local scale and the few cross-site experiments that manipulated animals (Heneghan *et al.*, 1999; Gonzalez & Seastedt, 2001;) are restricted by the low number of sites, differences in litter types and qualities between experiments, and the fact that due to a vast and mostly unknown biodiversity in many biomes, invertebrate analyses have been typically restricted to one to three taxonomic groups.

The Global Litter Invertebrate Decomposition Experiment (GLIDE) tests the hypothesis that soil animals significantly influence litter decomposition rates, over and above climate alone, at regional and global scales. A second hypothesis, related to the first and following Swift *et al*. (1979), is that the role of animals in terrestrial decomposition changes from region to region rather than having a single global character. The centerpiece of GLIDE's design was the use of a single plant litter substrate, of known quality and from a single origin, which was exposed in standard litterbags while manipulating the abundance and richness of soil fauna with a generalized suppressor (naphthalene). Decomposition was monitored in both animal-suppressed bags and untreated controls by gravimetric measurement and chemical analysis at 30 sites distributed across broad climatic regions from 43°S to 68°N (Table 1). Additionally, the extraction and characterization of associated (mainly mesofaunal) invertebrate animals was assessed. The use of the faunal-suppressant separated climate from animal effects on litter decomposition. Animal data, with complementary information on local climate and the weight and carbon content of litter at the start of the decomposition process and after exposure in the field, show that soil animals positively influence decomposition rates in the temperate and wet tropical biomes.

**Table 1:** GLIDE site descriptions, GLIDE climatic regions, and annual CDI values

Country	Site code	Latitude Longitude	Soil type	Vegetation type	Holdridge Life Zone	GLIDE climate region	Precipitation (cm)	Temperature (°C)	Annual CDI
Australia	TAS1	43°04′S 146°40′E	Planosol	Callidendrous fern rainforest	Cool temperate moist forest	Temperate	110.1	8.8	0.2711
Australia	TAS2	43°04′S 146°40′E	Acrisol	Intermediate rainforest	Cool temperate moist forest	Temperate	110.1	8.8	0.2711
Brazil	MAN	02°25′S 60°00′W	Ferralsol	Tropical moist forest	Tropical rain forest	Wet tropical	211.1	26.3	0.8311
Brazil	RUB	19°30′S 42°30′W	Ferralsol, Acrisol	Tropical rainforest	Subtropical rain forest	Wet tropical	116.8	22.7	0.4881
Burkina Faso	BON	11°20′N 4°15′W	Luvisol	Shrub to wooded savanna	Tropical dry forest	Dry tropical	95.7	27.1	0.4150
Canada	INU	68°19′N 133°32′W	Gleysol	Black spruce, paper birch forest	Subpolar dry tundra	Cold or dry	17.3	−11.3	0.0443
Canada	YUK	60°51′N 135°12′W	Luvisol	Pine, spruce, aspen forest	Boreal moist forest	Cold or dry	26.5	−4.2	0.0583
Canada	TOP	54°36′N 126°18′W	Luvisol	Pine, balsam, fir, spruce forest	Cool temperate moist forest	Cold or dry	48.8	2.1	0.1071
Canada	LET	50°11′N 113°54′W	Chernozem	Fescue grassland	Cool temperate steppe	Cold or dry	44.3	4.4	0.1477
Canada	VAN	48°38′N 123°42′W	Cambisol	Douglas fir forest	Cool temperate wet forest	Temperate	196.0	6.2	0.2287
Canada	ROC	49°32′N 58°49′W	Podzol	Balsam fir, white birch forest	Boreal wet forest	Temperate	115.2	1.6	0.2287
Canada	ONE	49°01′N 110°23′W	Chernozem	Shortgrass	Cool temperate steppe	Cold or dry	36.0	3.9	0.1080
China	XIS	21°41′N 101°25′E	Acrisol	Tropical seasonal rainforest	Subtropical moist forest	Wet tropical	132.2	20.5	0.5275
Colombia	BOG	04°37′N 74°18′W	Andosol, Cambisol	Montane cloud forest	Tropical wet forest	Dry tropical	117.5	14.7	0.4288
Czech Rep.	BIO	48°42′N 16°49′E	Fluvisol	Riparian oak forest	Cool temperate moist forest	Temperate	54.0	9.3	0.2462
Czech Rep.	SUM	49°97′N 13°45′E	Cambisol	Mountain climax spruce forest	Cool temperate moist forest	Temperate	64.8	7.6	0.2537
Czech Rep.	KOM	48°42′N 16°49′E	Fluvisol	Riparian oak forest	Cool temperate moist forest	Temperate	54.0	9.3	0.2462
Czech Rep.	PAL	48°42′N 16°49′E	Rendzina	Xerothermic grass/deciduous forest	Cool temperate steppe	Temperate	54.0	9.3	0.2462
Germany	GIE	51°20′N 10°22′E	Luvisol	Temperate beech forest	Cool temperate moist forest	Temperate	68.3	8.1	0.2618
Germany	JEN	50°57′N 11°35′E	Rendzina	Xerothermic grassland	Cool temperate steppe	Temperate	58.6	8.2	0.2432
Kenya	KEN	0°15′S 34°50′E	Acrisol	Rainforest	Tropical wet forest	Wet tropical	185.6	20.7	0.6406
Malaysia	MAL	05°47′N 116°24′E	Acrisol	Tropical lower montane forest	Subtropical wet forest	Wet tropical	260.7	22.8	0.7587
Namibia	NAM	23°33′S 15°02′E	Gleysol, Histosol	Sparse dwarf shrub	Subtropical desert	Cold or dry	0.1	23.5	0.0264
Russia	KUR	51°34′N 36°05′E	Chernozem	Oak forest, meadow steppe	Cool temperate steppe	Temperate	65.2	5.5	0.2253
Senegal	SEN	16°25′N 15°30′W	Arenosol	Dry savanna, balanites	Tropical desert scrub	Dry tropical	26.1	27.1	0.1565
Taiwan	TAI	24°46′N 121°35′E	Acrisol, Cambisol	Evergreen, hardwood forest	Subtropical moist forest	Wet tropical	208.2	20.6	0.7313
UK	CAR	51°50′N 04°10′E	Cambisol	Oak forest	Cool temperate moist forest	Temperate	82.6	9.8	0.2961
UK	SIL	51°24′N 0°40′W	Cambisol	Oak, oak-birch, alder forest	Cool temperate moist forest	Temperate	74.1	9.3	0.2414
USA	COL	40°49′N 104°46′W	Yermosol	Shortgrass steppe	Cool temperate steppe	Cold or dry	38.5	8.7	0.1222
USA	FLO	27°09′N 81°12′W	Luvisol, Histosol	Native tallgrass wet prairie	Subtropical moist forest	Wet tropical	127.4	22.7	0.6060

GLIDE refers to the Global Litter Invertebrate Decomposition Experiment. Soil type is a UN FAO classification. Vegetation type is the description provided by the 30 GLIDE site collaborators (see following names). Holdridge Life Zone classification (Leemans, 1990) is determined by latitude and longitude coordinates. Precipitation and temperature values are long-term averages based on climate data gathered from weather stations nearest to each study site. CDI is the Climate Decomposition Index (Parton *et al.*, 2007). We categorized sites into four climatic regions based on site vegetation and abiotic data, with wet and dry tropical sites considered separately, based on precipitation and abiotic models (Schimel *et al.*, 1996). Site codes (see Table 1) location, and collaborators are as follows: TAS1 and TAS2, Tasmania, Warra Long Term Ecological Research (LTER), R. Bashford; MAN, Manaus, H. Vasconcelos; RUB, Rubro Negra, M. Sabará; BON, Bondoukuy, D. Masse; INU, Inuvik, NT, Canadian Intersite Decomposition Experiment (CIDET), L. Kutny; YUK,Whitehorse, YT, CIDET, D. White; TOP, Topley, BC, CIDET, M. Kranabetter; LET, Stavely, AB, D. Johnson; VAN, Shawnigan, Vancouver Isl., BC, CIDET, J. Trofymow; ROC, Rocky Harbour, NL, CIDET, S. Flemming; ONE, Onefour, AB, D. Johnson; XIS, Xishuangbanna Tropical Botan. Grdn., X. Zou; BOG, Bogotá, A. Varela; BIO, Palava Biosphere Reserve South Moravia, J. Rusek; SUM, Sumava National Park, M. Kovarova; KOM, Palava National Park 2, J. Rusek; PAL, Palava National Park 1, J. Rusek; GIE, Giessen, J. Salamon; JEN, Jena, W. Voigt; KEN, Kakamega Forest, M. Swift; F. Ayuke; MAL, Tambunan, M. Maryati and R. Homathevi; NAM, Gobabeb, J. Henschel; KUR, Central Chernozem Reserve, A. Pokarzhevskii O. Beljakova; SEN, Souilene, A. Brauman; TAI, Fu-shan Taiwan Ecological Research Network, K. Lin; CAR, Cardiff, Wales, T. Jones; SIL, Silwood Park, London, M. Bradford; COL, Colorado, Shortgrass Steppe LTER, D. Wall; FLO, Florida, MacArthur Agro-Ecolog. Res. Center, P. Bohlen. Contacts for 11 GLIDE sites not included in this analyses are as follows: Australia (one site), T. Adams; Brazil (one site), D. da Motta Marques; Beijing (one site), Y. Wang; Israel (one site), Y. Steinberger; Mongolia (two sites), R. Baatar; Mongolia (three sites), Sh. Tsooj; Poland (one site), M. Sterzynska; Venezuela (one site), A. Torres Lezama.

## Material and methods

### Site management and location

Forty-one sites were initially established in 2001–02, as part of the DIVERSITAS IBOY (International Biodiversity Year) Project network, and with ILTER (International Long Term Ecological Research) collaborators. Sites were selected to achieve the fullest practical climatic and geographic range, but partially reflected the mostly volunteer participation by national science teams in IBOY. Eleven sites were dropped from analyses due to destruction of bags *in situ*, excessive extraneous organic matter or soil in the litterbags, or where export of specimens from countries was prohibited or impractical. The 30 remaining sites are described in Table 1.

### Experimental design

Two thousand glass-fiber, 20 cm × 20 cm, 2 mm mesh litterbags (Harmon *et al.*, 1999), were each filled with 10±0.5 g dried, gamma-sterilized grass hay [*Agropyron cristatum* (L.) Gaertn.] foliar litter that had been air-dried for 2 years at <20% rH. The litter, with all florets removed, was preprocessed through a 1.0 mm screen (to remove loose material before shipping) and then shipped from Colorado State University (CSU) in the constructed glass-fiber bags to sites. A single litter quality was selected to facilitate site-to-site comparisons and because the scale of resources required to analyze all taxa from just one litter type made multilitter field experiments unfeasible. Bags were weighed on-site to the nearest 0.001 g before being secured to the soil surface, and then progressively removed over time intervals. Of 24 bags at each site, six were placed randomly in each of four experimental blocks. Within each block, three bags were spaced along each of two paired 20 m transects, with each transect being assigned to either the control or animal-suppressed treatment. Transects ran parallel to each other and were 10 m apart. The three bags within each transect were spaced at 0, 10, and 20 m. All blocks were at least 10 m apart, and positioned randomly. This design was chosen to (a) ensure the suppression treatment did not influence the control litterbags, (b) reduce the impacts of disturbance by vertebrates, and (c) reduce the effects of spatial autocorrelation.

### Naphthalene treatment: animal suppression

The animal inhibitor naphthalene was added to half of the litterbags; the other half was assigned to untreated controls. Naphthalene was applied at the start of the experiment in crystalline form (as ‘mothballs’ from a single commercial source) and again at each sampling occasion to litterbags that remained in the field for later retrieval. For each treatment renewal, two mothballs (∼33.17 g per mothball) were placed adjacent to treatment bags. Naphthalene was chosen to displace soil animals because it reportedly has less biocidal effects than other pesticides (Blair *et al.*, 1989). An additional three ‘traveler bags’ were shipped to each site, placed in the field, immediately retrieved, reweighed, and reshipped to CSU for analysis. Traveler bags controlled for material loss due to handling (Harmon *et al.*, 1999). See also http://www.nrel.colostate.edu/projects/glide/studydesign.html.

### Litterbag retrieval and processing

One control and one treatment litterbag were retrieved from each block on three sampling occasions. Time of field exposure was dependent on climate, longer where litter decomposition rates were slower due to high latitude (e.g. temperate sites) or high altitude (e.g. BOG site; Table 1). Three Canadian sites were sampled at 2 and 12 months, and another two were sampled at 2 and 4 months only (Table 2). Soil animals were extracted from bags at sites by a standardized Tullgren dry heat apparatus shipped from CSU. Animals were shipped in 70% ethanol to BioTrack® Australia Pty Ltd (Macquarie University, Sydney) for centralized taxonomic identification. Voucher specimens were stored until investigators requested return. After weighing, fauna-extracted litterbags were returned to CSU where subsamples of oven-dried, plant material were milled and analyzed for C on a LECO 1000 CHN analyzer (LECO Corp., St Joseph, MI, USA). Initial litter C concentration was 44.13%. The composition of the initial litter ‘lignin’ (Preston *et al.*, 1997, 2006) was as follows: neutral detergent fiber or NDF extract, 67.91% (SD±0.74); acid hydrolyzable extract, 27.54% (SD±0.83); acid unhydrolyzable residue or AUR, 4.14% (SD±0.18); and ash, 0.41% (SD±0.44). ADF, acid detergent fiber, is the sum of the last three measures.

**Table 2:** GLIDE (Global Litter Invertebrate Decomposition Experiment) climatic region, sampling intervals, and analyses performed for each site

Laboratory analyses						Sampling times (months)				
Climatic region treatment	GLIDE site code	Min. FRC (%)	*k* (day^−1^)	*kr*^*2*^	Taxonomic richness	1	2	3	4	12
Cold or dry	INU				X		X			X
Naphthalene		45.31	−0.00077	0.76						
No naph		42.51	−0.00080	0.87						
Cold or dry	YUK				X		X			X
Naphthalene		46.52	−0.00077	0.95						
No naph		49.96	−0.00075	0.91						
Cold or dry	TOP				X		X			X
Naphthalene		33.59	−0.00105	0.88						
No naph		39.61	−0.00096	0.77						
Cold or dry	LET				X		X		X	
Naphthalene		56.39	−0.00175	0.98						
No naph		57.33	−0.00185	0.99						
Cold or dry	ONE				X		X		X	
Naphthalene		48.54	−0.00225	0.99						
No naph		45.04	−0.00257	0.95						
Cold or dry	NAM						X		X	X
Naphthalene		69.58	−0.00036	0.91						
No naph		67.52	−0.00045	0.95						
Cold or dry	COL				X		X		X	X
Naphthalene		58.74	−0.00056	0.97						
No naph		60.23	−0.00055	0.95						
Temperate	TAS1						X		X	X
Naphthalene		43.76	−0.00088	0.61						
No naph		32.99	−0.00130	0.57						
Temperate	TAS2						X		X	X
Naphthalene		42.13	−0.00114	0.77						
No naph		37.20	−0.00115	0.71						
Temperate	VAN				X		X		X	X
Naphthalene		33.44	−0.00127	0.83						
No naph		28.59	−0.00146	0.86						
Temperate	ROC				X		X		X	X
Naphthalene		38.02	−0.00129	0.81						
No naph		26.25	−0.00139	0.82						
Temperate	BIO				X		X		X	X
Naphthalene		23.97	−0.00188	0.85						
No naph		13.36	−0.00224	0.93						
Temperate	SUM				X		X		X	X
Naphthalene		37.48	−0.00120	0.82						
No naph		43.75	−0.00114	0.78						
Temperate	KOM				X		X		X	X
Naphthalene		6.35	−0.00290	0.95						
No naph		5.45	−0.00338	0.98						
Temperate	PAL				X		X		X	X
Naphthalene		17.85	−0.00204	0.89						
No naph		7.81	−0.00260	0.95						
Temperate	GIE				X		X		X	X
Naphthalene		24.48	−0.00163	0.98						
No naph		23.39	−0.00182	0.94						
Temperate	JEN						X		X	X
Naphthalene		20.39	−0.00203	0.90						
No naph		18.71	−0.00189	0.89						
Temperate	KUR						X		X	X
Naphthalene		27.60	−0.00150	0.87						
No naph		30.97	−0.00151	0.85						
Temperate	CAR						X		X	X
Naphthalene		32.55	−0.00156	0.79						
No naph		23.5	−0.00196	0.73						
Temperate	SIL						X		X	X
Naphthalene		23.83	−0.00193	0.78						
No naph		10.53	−0.00245	0.90						
Wet tropical	RUB					X	X	X		
Naphthalene		23.21	−0.00579	0.91						
No naph		9.53	−0.00737	0.90						
Wet tropical	MAN					X	X	X		
Naphthalene		22.69	−0.00737	0.97						
No naph		11.82	−0.00853	0.79						
Wet tropical	XIS				X	X	X	X		
Naphthalene		19.66	−0.00838	0.93						
No naph		19.77	0.00823	0.90						
Wet tropical	KEN				X	X	X	X		
Naphthalene		61.33	−0.00143	0.45						
No naph		39.06	−0.00376	0.78						
Wet tropical	MAL				X	X	X	X		
Naphthalene		17.36	−0.00752	0.98						
No naph		24.60	−0.00619	0.91						
Wet tropical	TAI					X	X	X		
Naphthalene		19.40	−0.00653	0.75						
No naph		9.25	−0.00887	0.66						
Wet tropical	FLO				X	X	X	X		
Naphthalene		30.24	−0.00349	0.59						
No naph		31.78	−0.00514	0.91						
Dry tropical	BON					X	X	X		
Naphthalene		5.71	−0.00891	0.78						
No naph		20.45	−0.00562	0.76						
Dry tropical	BOG				X		X		X	X
Naphthalene		0.54	−0.00581	0.98						
No naph		0.10	−0.00679	0.96						
Dry tropical	SEN					X	X	X		
Naphthalene		3.99	−0.00706	0.62						
No naph		26.20	−0.00501	0.96						

Fraction remaining carbon of litter (FRC) reported is for the final sampling date – note that values are only comparable across sites where the final sampling times are the same; *r*^*2*^ is calculated for the exponential fit used to derive the *k-*value (litter decomposition rate). Sites organized alphabetically by country (see Table 1 for site code explanation). Sampling dates and taxonomic richness analyses performed for each site are designated by X.

### Taxonomic characterization

Taxonomy was determined according to The Tree of Life (http://www.tolweb.org/tree/) and Systema Naturae 2000 (http://sn2000.taxonomy.nl/). BioTrack® (Harvey & Yen, 1990; Oliver *et al.*, 2000;) identified all animals, except for Czech Republic sites BIO, KOM, PAL, and SUM; Brazilian sites MAN and RUB; and Colombian site BOG (Table 1) where taxonomy was carried out by qualified experts. All adult animals extracted from litterbags were identified to 38 invertebrate groupings (Table 3) with Pauropoda and Symphyla identified to Class, mites (Acari) identified to Subclass, and other taxa identified to the Order level. The number of Orders ranged from one each in Phyla Annelida and Mollusca, two in Class Crustacea, four in Subclass Arachnida (Acari considered one ‘Order’), 11 in Class Myriapoda (Pauropoda and Symphyla considered as ‘Orders’), to 19 in Class Hexapoda.

**Table 3:** Taxa of invertebrates identified in GLIDE (Global Litter Invertebrate Decomposition Experiment) litterbags

Phylum	Subphylum	Class	Subclass	Order
Arthropoda	Crustacea	Malacostraca	Peracarida	Isopoda Amphipoda
	Hexapoda	Entognatha		Diplura
		Entognatha	Protura	Eosentomata
		Entognatha		Collembola
		Insecta	Archaeognatha	Microcoryphia
			Pterygota	Blattodea Coleoptera Dermaptera Diptera Embioptera Hemiptera Hymenoptera Isoptera Lepidoptera Megaloptera Neuroptera Orthoptera Psocoptera Thysanoptera Trichoptera
	Myriapoda	Diplopoda	Helminthomorpha	Spaerotheriida Chordeumatida Julida Spirobolida Spirostreptida Polydesmida
			Penicillata	Polyxenida
		Chilopoda	Pleurostigmophora	Geophilomorpha Lithobiomorpha
		Pauropoda		
		Symphyla		
	Chelicerata	Arachnida	Acari	
			Micrura	Araneae
			Dromopoda	Opilionida
				Pseudoscorpionida
Annelida	Clitellata	Oligochaeta		Haplotaxida
Mollusca	Gastropoda	Orthogastropoda	Pulmonata	Stylommatophora

Tree of Life (http://www.tolweb.org/tree/phylogeny.html), accessed on February 11, 2008.

Systema Naturae 2000 (http://www.taxonomy.nl/Main/Classification/1.htm), accessed on February 11, 2008. Taxa listed under Class and Subclass, but not under Order, were not identified to the Order level.

### Statistical analyses

Biota (Colwell, 2004), a relational database application, was used to manage data. Of four litterbags removed per treatment (control and suppression treatment) per sampling period at each site, two were for determination of C concentrations of the residual litter. Knowing the initial- and post-field-exposure litter masses, and the initial and retrieved litter C concentrations, the log_e_ of the fraction remaining litter C (FRC) was regressed against the number of field days of litter exposure and *k* was determined from the slope as an estimate of litter decomposition rate (Harmon *et al.*, 1999). By using FRC rather than fraction litter mass remaining *per se*, we could correct for incorporation of mineral material into litterbags, a procedure similar to the use of ash-free dry mass (Harmon *et al.*, 1999).

**Fig. 1:**
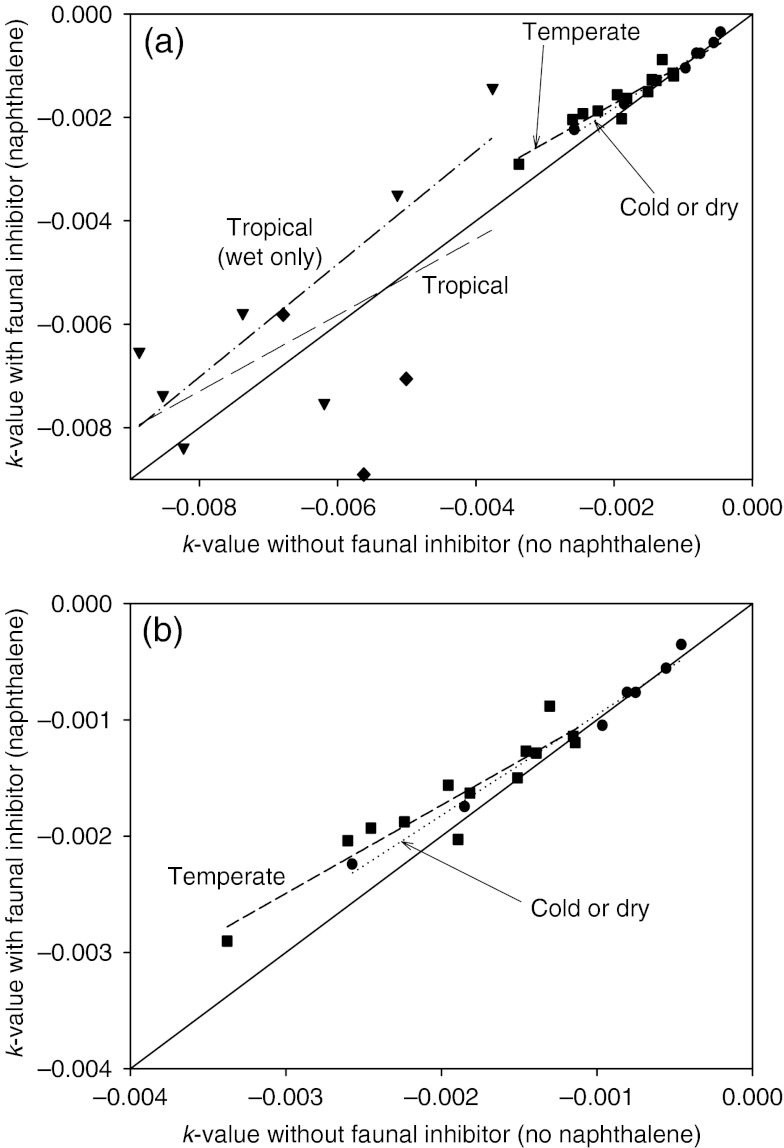
Impact of the animal-suppressant naphthalene on litter decomposition rate (*k*) at climatic sites. (a) Departures (represented by broken lines for each climatic region) from the 1 : 1 solid line represents an impact of the animal suppressant on decomposition rates: values above the line indicate lower decomposition rates when the suppressant was present and vice versa. Data for 30 sites and four climatic regions (Table 1) are shown: cold or dry (circles), temperate (squares), wet tropical (triangles), dry tropical (diamonds). (b) Data for the cold or dry (circles) and temperate (squares) sites are replotted to clearly show the departure from the 1 : 1 line for the temperate region.

For each of the 30 sites, the *k*-value for the inhibitor treatment bags was regressed against the *k*-value for control bags (Fig. 1), deviations from the 1 : 1 line being associated with potential treatment effects. Paired (by site) *t*-tests were performed using untransformed *k*-values and one-tailed tests, given that variance was determined to be equal and the hypothesis that animal inhibitors would only decrease decomposition rates respectively. These tests were first performed for all sites together and, second, to determine if there were region-specific effects, for sites within each climatic region.

**Fig. 2:**
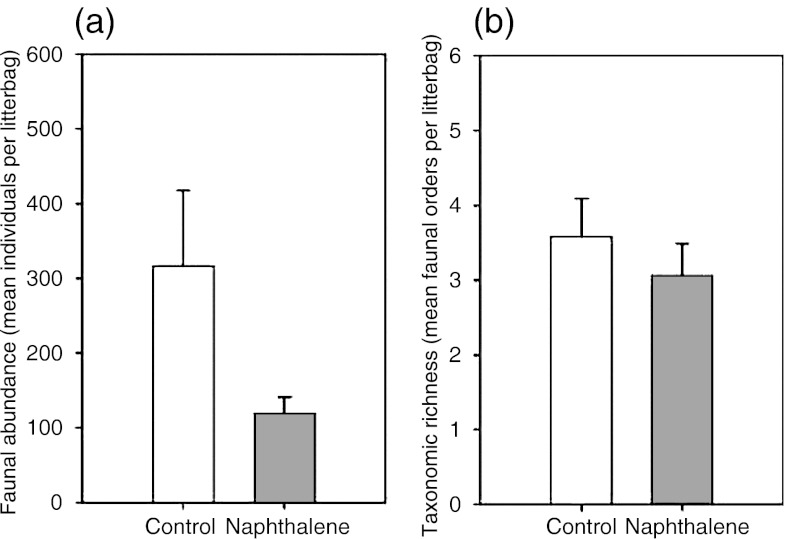
Impact of the animal-suppressor naphthalene on animal diversity in litterbags. (a) Animal abundance in the control (open bars) and suppressant (filled bars) treatment. (b) Animal taxonomic richness.

For 18 of our 30 sites, we had complete animal abundance and taxonomic richness data (Tables 2 and 3) for each of the eight litterbags removed on each sampling occasion. The other 12 sites had missing values for some sample times. For the 18 sites with complete datasets, mean abundance and richness were determined for control and animal-suppressed treatments at each sampling period, and then mean abundance and richness across all sampling times. This gave single animal abundance and taxonomic richness values for both the control and animal-suppressed treatments for each site. Effects of the animal inhibitor on animal abundance and taxonomic richness were tested using, as for the *k* data, one-tailed paired *t*-tests (Fig. 2). Unequal variance in abundance data was equalized by natural log transformation.

To test whether the impacts of the animal suppressor on litter decomposition rates were correlated with treatment effects on animal abundance and/or taxonomic richness, we used multiple linear regression and regression tree approaches (Crawley, 2002). The primary reason for conducting these analyses was to evaluate whether the expected statistical relationship between decomposition rate and climate was altered by changes in the abundance and/or diversity of fauna. In both regression analyses, decomposition rate (*k*) was used as the response variable, natural log-transformed to meet assumptions of homogeneity of variance. There were three explanatory variables: mean animal taxonomic richness, mean animal abundance, and the function ‘climate decomposition index’ (CDI; Table 1). The influence of climate on decomposition is usually represented in global decomposition models as a function of temperature and water availability (Liski *et al.*, 2003; Parton *et al.*, 2007;). For analysis of our data, we used the function CDI, a widely recognized index for predicting climate effects on litter decomposition (Moorhead *et al.*, 1999; Gholz *et al.*, 2000;) and an integral part of the global carbon model CENTURY (Parton, 1996). An annual CDI value for each site was calculated from monthly values for precipitation and temperature (Tables 1 and A1) (Parton *et al.*, 2007). The temperature function uses average monthly maximum and minimum air temperatures and has been validated on an extensive global soil respiration dataset (Del Grosso *et al.*, 2005). The water stress term is calculated as a function of the ratio of monthly rainfall to potential evapotranspiration rate (Parton *et al.*, 2007). For GLIDE, average CDI values were calculated for the period that corresponded to the maximum sampling time achieved (e.g. 12 months for temperate sites) (Table 1). Monthly values of maximum and minimum air temperature and precipitation data from weather stations close to sites were used (see Appendix) because there were incomplete data at all sites for these years.

A total of 35 values were used for regression analyses, from the subset of 18 sites for which complete animal data were available. These comprised two sets of animal variables (richness, abundance), one from each treatment (control, animal-suppressed). Through model checking, one point (Kenya, animal-suppressed litterbag) was omitted because it consistently violated assumptions of homogeneity of variance irrespective of the transformations used.

For regressions using CDI, richness, and abundance values, the simplest possible model that included all three explanatory variables was fitted first. Both *k* and CDI were plotted as natural log-transformed variables. Next, the curvature was tested by fitting the quadratic terms for the variables; a square-root transformation of the abundance was found necessary to correct for curvature.

## Results

### Soil animal diversity

The 2 mm litterbag mesh prevented the access of soil animals greater than 2 mm in diameter, excluding larger animals. Some macrofauna (e.g. spiders, termites, beetles, and other insects) could gain access as immatures, but of the 80 606 individuals sorted to 38 higher taxonomic groupings (i.e. Class, Subclass, Order), the majority were soil mesofauna (defined as having maximum body widths ranging from 100 μm to 2 mm as adults; Swift *et al.*, 1979) (Table 3).

### Latitudinal effects of animal suppressant naphthalene

The *k*-values from the 30 sites (Table 1) were used to test whether the naphthalene reduced litter decomposition rates. For each site, the *k*-value for the animal-suppressed treatment bags was plotted against the *k*-value for control bags (Fig. 1), deviations from the 1 : 1 line being associated with potential treatment effects. In some climate regions, the inhibitor had a negative effect on *k*.

Across all 30 sites, treatment with naphthalene did not affect litter decomposition rates (*k*-value, Fig. 1a). When the same analyses were performed for each of our four climatic regions (Table 1), decomposition rates were significantly reduced by the animal suppressant in temperate and wet tropical regions (temperate, *t*_1,12_=3.6, *P*<0.01; wet tropical, *t*_1,6_=2.1, *P*<0.05), but not in cold or dry regions, or the tropics as a whole (*P*>0.05 for all three datasets) (Fig. 1a and b). To corroborate that these negative and neutral effects on decomposition rates were associated with animal responses to the suppressant, we evaluated whether naphthalene decreased the abundance and/or mean taxonomic richness (Table 3) of animals extracted from litterbags. When the suppressant was present, there were significant reductions in both abundance (*t*_1,17_=1.8, *P*<0.05) and richness (*t*_1,17_=2.8, *P*<0.01). Log-transformed abundance and taxonomic richness were found to be correlated but weakly, using Pearson's product-moment statistic (*R*^2^=0.376, *t*_34_=4.5, *P*<0.01, *n*=18 sites). Treatment values (means±1 SE) for each site were calculated as the mean of all three sample periods (Fig. 2a and b).

### Animal effects on the relationship between climate and litter decomposition

**Fig. 3:**
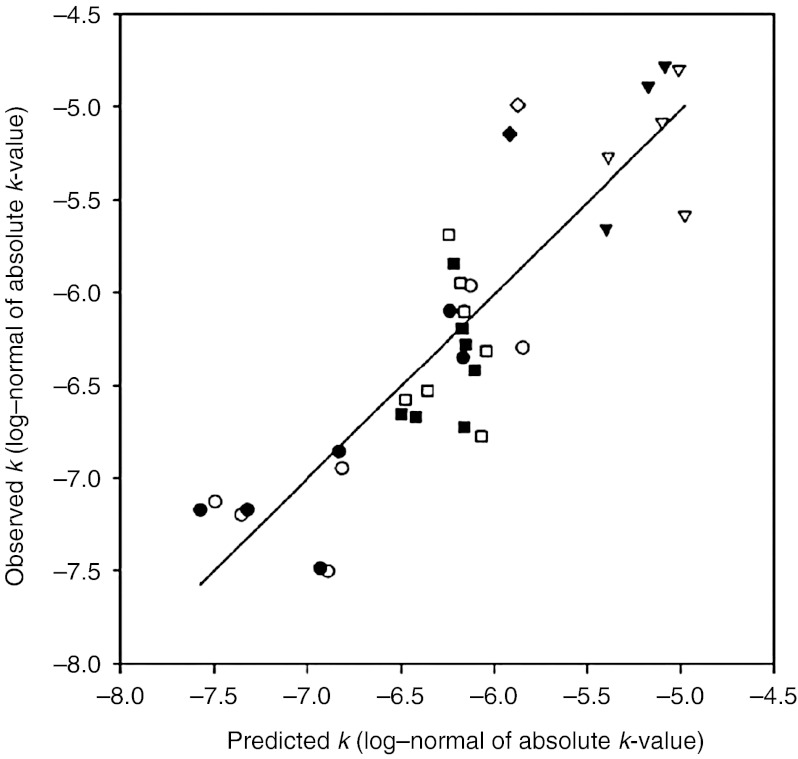
Fit of the minimal adequate model of decomposition rate (*k*). In both these approaches, determined by step-wise multiple linear regression to observed values, and using a regression tree analysis, climate, as modeled using the temperature and water availability climate function climate decomposition index (CDI) (see ‘Material and methods’), and soil animal taxonomic richness were retained as significant explanatory variables for decomposition rate (*k*). Symbols for each climatic region (Table 1) are as follows: cold or dry (circles), temperate (squares), wet tropical (triangles), dry tropical (diamonds). Filled symbols represent the inhibitor treatment while open symbols represent control.

Analysis of covariance was used to test whether the global CDI–decomposition relationship was influenced by the presence of naphthalene. The interaction between the CDI and suppressant terms was not significant (*P*>0.05), suggesting that the global relationship between climate and litter decomposition rates is robust to experimental reductions in soil animal abundance and taxonomic richness. The animal-suppressed treatment was then substituted for mean animal abundance and mean animal richness data. These variables could be used along with CDI annual values (Tables 1 and A1) as continuous explanatory variables, providing 35 independent data points for regression analyses (see ‘Material and methods’). That is, the subset of 18 sites (Table 2) for which complete animal abundance and taxonomic richness data were available (Table 3) was used for the regression. This confounds the animal and climate effects and therefore requires cautious interpretation (Loreau *et al.*, 2002; Wardle, 2002;); this limitation is more fully evaluated in ‘Discussion’. The minimal adequate model (log_e_[*k*-value]=0.1271[taxonomic richness]+0.6299 × log_e_[CDI]−5.755) obtained using step-wise multiple linear regression retained CDI and taxonomic richness as explanatory variables and accounted for 77% of the variation in decomposition rates [*P*<0.001 (*F*_2_,_32_=53; *n*=35)] (Fig. 3).

Given that CDI alone explained 70% of the variation in measured decomposition rates, as shown in other multisite global decomposition experiments, the validity of designating fauna as an explanatory variable was further tested using a regression tree approach (results not shown). This confirmed that animal abundance *per se* offered little explanatory power, but that taxonomic richness of soil fauna, in addition to climate, was correlated with decomposition rates across broad climatic regions.

## Discussion

GLIDE provides the first high-resolution taxonomic database relating soil fauna to decomposition on a global scale. It improves upon pairwise comparisons of temperate vs. tropical regions (Heneghan *et al.*, 1999; Gonzalez & Seastedt, 2001;) to include a gradient of climatic conditions from 30 sites globally, and improves taxon breadth from three or less to 38 taxonomic groups. The findings of this experiment indicate that soil mesofaunal assemblages, dominated by arthropods, influence litter decay across broad climatic regions, namely temperate and wet tropical, as predicted by Swift *et al*. (1979). If this applies to all soil invertebrates, the original conceptual model of three primary drivers of litter decomposition (climate, litter quality, and biota; Swift *et al.*, 1979) is validated.

Investigations using biogeographical comparisons to explore linkages between ecosystem processes and organism diversity are typically frustrated by the positive correlation between diversity and the product of temperature and water availability (Coûteaux *et al.*, 1995; De Deyn & Van der Putten, 2005;), though Maraun *et al*. (2007) have offered oribatid mites, one of the most abundant and species-rich groups of soil mesofauna, as an exception to this rule. In our study, we overcame this limitation by experimentally reducing animal richness and abundance at each site independently of climate, an approach already used effectively in smaller scale, cross-site experiments (Heneghan *et al.*, 1999; Gonzalez & Seastedt, 2001;). By using an analysis of covariance (as opposed to regression approaches) in this initial analysis, we ensured that our statistical tests did not confound the animal and climate effects (Loreau *et al.*, 2002; Wardle, 2002;). One potential limitation of the approach, however, is that naphthalene may negatively affect other components of soil assemblages. Under field conditions, however, naphthalene tends to have negligible effects on soil bacterial and fungal growth (Blair *et al.*, 1989) and is relatively persistent. Using this approach, we demonstrate that soil fauna positively influence decomposition rates in the temperate and wet tropical biomes and are neutral in regions where biological activity is more constrained by temperature and/or moisture, which was hypothesized by Swift *et al*. (1979) but untested until now.

Our regression analyses, where the categorical factor naphthalene was substituted for the continuous variables of abundance and richness, requires cautious interpretation because of the confounding influence of climate once this statistical approach is used. That is, in contrast to the categorical factor naphthalene, abundance and richness may be correlated with temperature and moisture (i.e. climate), and so relationships between abundance and richness with decomposition rates may be artefacts of this correlation. It is, however, noteworthy that taxonomic richness of soil fauna, and not abundance, was positively related to litter decomposition rates. In contrast, experimental evidence from microcosm and local-scale field experiments suggests that it is animal abundance or biomass, and not richness, which operate as the driver (Cole *et al.*, 2004). Our experiment did not allow for the determination of animal biomass *per se*, so we cannot discount the possibility that the effect of taxonomic richness was driven by biomass. The key difference between our experiment and previous investigations into relationships between soil animal diversity and litter decomposition rates, aside from geographic scale, is that we identified the entire extracted animal assemblage in our litterbags to Class, Subclass or Order, rather than identifying only a few select groups to species (Table 3). The high species richness of soil animals is assumed to be associated with high functional redundancy (Andrén & Balandreau, 1999), whereas animal richness at the Order level or higher is concluded to be associated with higher functional dissimilarity (Naeem & Wright, 2003; Heemsbergen *et al.*, 2004; St. John *et al.*, 2006;); that is, the greater the value of taxonomic richness at this level, the more functionally diverse the animal assemblage. However, this concept is difficult to test in the field because no single soil or litter decomposition study has had the resources to analyze all species of soil fauna present at one time, owing to the expertize required for the accurate identification of each group and the high percentage (∼85%) of soil fauna yet to be described (Eggleton & Bignell, 1995; Lawton *et al.*, 1998; St. John *et al.*, 2006;). The present study, utilizing the BioTrack® facilities, takes advantage of recent developments in semiautomated methods of rapid biodiversity assessment (Oliver *et al.*, 2000) and suggests that functional richness of soil fauna may be an important driver of decomposition across large geographic regions. Further work will be required to test this conclusion definitively. We recommend that additional studies should address fewer representative sites, but with greater replication per site, higher taxonomic resolution, biomass determination, and the use of robust litterbags excluding mesofauna and macrofauna as an additional suppression treatment augmenting naphthalene. The use of multiple litter species of differing qualities should also be considered, given that faunal effects may be quality dependent. A more recalcitrant litter than used in our study, decomposing for a longer time, would likely involve a different biodiversity and abundance and perhaps show other effects on decomposition rates. However, the effort required for this and the other improvements to the field procedure is very large.

**Fig. 4:**
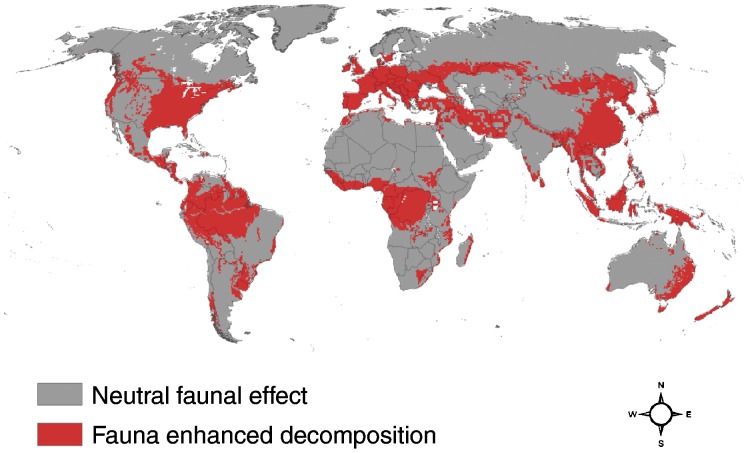
Map showing climatic regions where soil animals enhance decomposition rates. Results from temperate and wet tropical climatic regions (red) show fauna increase decomposition rates, but have neutral effects in other regions (gray). This suggests that in future scenarios of climate change for regions predicted to be warmer and wetter, enhanced decomposition rates may result from soil animals and other biota. Results are based on Global Litter Invertebrate Decomposition Experiment (GLIDE) climatic regions and sites presented in Table 1 and the table in the Appendix (Table A1). The location of climatic regions shown here is based on the potential natural vegetation system used in the VEMAP analyses (VEMAP, 1995).

It is unlikely that potential nontarget effects of the suppressant on microbial activity explain the results in the field, because inhibitor effects were restricted to specific climatic regions. If nontarget effects had occurred, then litter decomposition should have been retarded in all climatic regions because the climate driver in decomposition models (CDI) is a surrogate for bacterial and fungal activity *per se*. Therefore, soil mesofauna, likely through their functional or taxonomic richness, are an important driver of litter decomposition rates in some climatic regions. When these regions are viewed at a global scale under current climatic conditions (Fig. 4), they account for 27.2% of the Earth's land area.

These results indicate that explicit inclusion of soil animals in decomposition models may reduce the unexplained variation in relationships between litter decomposition and climate across regional scales. We found evidence that inclusion of soil animals (specifically, their diversity) in models of global-scale decomposition rates may provide modest improvements in predictability [from 70% to 77% variance explained over abiotic factors (CDI) alone] but the underlying mechanism for this remains unclear and warrants further study. Notably, however, our finding that animals have important influences on decomposition in certain climatic regions has potential implications for global carbon dynamics under changing climate, and may help explain regional variability in soil respiration (Davidson *et al.*, 2006). For example, regions predicted to have warmer and wetter climates may see positive feedbacks to soil communities, resulting in accelerated decomposition rates and release of carbon. In higher latitude systems, this feedback could substantially increase respiration of C to the atmosphere given the greater proportions of relatively undecomposed, organic carbon that has accumulated due to historically cold and dry conditions (Lal, 2005). Alternatively, global changes such as land use conversion (Swift *et al.*, 1998; Kaiser, 2004;) may alter soil animal diversity and/or biomass with consequences for decomposition rates in warm, wet climatic regions. Thus, the specific inclusion of regional impacts of soil animals on decomposition is necessary to adequately model the impacts of global change scenarios on decomposition and atmospheric C dynamics. Additionally, because litter quality may modify animal effects at a single site, future global experiments will need to test simultaneously all three primary drivers of decomposition.

## Appendix

Monthly values for mean precipitation (Precip, cm), maximum (*T*_max_) and minimum (*T*_min_) temperatures ( °C), and monthly climate decomposition index (CDI) calculation for each site. Italicised values indicate actual months in the field (see Table 2) for sites where litterbags were in the field for <12 months. For these sites only the italicised values were used to calculate the mean CDI. See Table 1 for explanation of site codes.

**Table A1:**

Site code	Variable	January	February	March	April	May	June	July	August	September	October	November	December
TAS 1 & 2	Precip	8.04	6.30	4.71	8.28	8.17	9.55	12.18	12.22	9.62	11.78	8.94	10.32
	*T*_max_	17.00	17.10	15.60	13.30	10.20	8.20	8.00	8.60	9.70	11.80	13.70	15.20
	*T*_min_	8.20	8.50	7.40	6.10	4.20	2.40	2.20	2.60	3.30	4.40	5.90	7.40
	CDI	0.3055	0.3223	0.2673	0.3177	0.2588	0.213	0.2077	0.2192	0.2411	0.2817	0.3048	0.3139
MAN	Precip	19.82	21.85	25.92	25.07	27.04	19.87	17.69	10.50	9.36	8.58	11.94	13.46
	*T*_max_	29.95	30.05	30.25	30.00	29.90	30.00	29.40	30.45	31.70	31.90	30.95	30.15
	*T*_min_	21.95	22.05	22.25	22.00	22.10	22.00	21.40	22.45	21.90	22.20	22.95	22.15
	CDI	0.8796	0.9023	0.9322	0.9283	0.9286	0.9277	0.9054	0.7639	0.6977	*0.5399*	*0.6748*	*0.8926*
RUB	Precip	19.14	10.56	13.41	6.01	3.61	1.45	0.93	1.98	6.16	9.83	19.48	24.20
	*T*_max_	29.93	30.15	29.85	28.78	27.13	25.75	25.13	26.15	26.88	28.08	28.55	28.68
	*T*_min_	19.93	20.15	19.85	18.78	16.13	13.75	13.13	14.15	15.88	18.13	19.15	19.63
	CDI	0.7624	0.6058	0.6773	0.4945	0.3025	0.1041	0.0529	0.1518	*0.3905*	*0.5827*	*0.8606*	0.8725
BON	Precip	0.03	0.03	1.48	3.88	9.14	13.60	19.63	26.02	15.23	5.61	0.88	0.21
	*T*_max_	32.30	35.60	36.60	36.40	34.60	31.60	29.50	28.00	29.80	32.30	33.70	32.70
	*T*_min_	17.30	20.00	23.60	25.40	24.60	22.20	21.50	21.20	21.40	21.50	19.70	18.10
	CDI	0.0296	0.0315	0.0831	0.1934	0.6064	0.8505	0.9154	0.8955	0.8907	*0.3882*	*0.0619*	*0.0338*
INU	Precip	0.86	0.83	1.00	0.78	1.28	1.35	2.22	2.71	2.34	1.94	1.07	0.96
	*T*_max_	−25.70	−24.80	−20.50	−12.80	−3.00	7.30	13.00	11.10	3.70	−4.70	−17.90	−23.30
	*T*_min_	−31.90	−31.60	−28.30	−21.40	−9.20	1.50	5.60	5.30	−0.30	−8.90	−23.90	−29.50
	CDI	0.0004	0.0005	0.0005	0.0014	0.0224	0.0354	0.0852	0.194	0.1414	0.0488	0.0005	0.0005
YUK	Precip	1.68	1.58	1.10	0.51	1.48	3.49	5.21	2.83	2.83	1.67	2.23	1.91
	*T*_max_	−14.30	−12.00	−4.80	2.60	9.00	13.80	16.00	14.80	8.70	0.30	−10.20	−11.90
	*T*_min_	−23.90	−22.80	−16.80	−8.20	−2.20	1.40	4.40	3.00	−1.50	−6.70	−17.80	−20.90
	CDI	0.004	0.004	0.0143	0.0111	0.0272	0.0839	0.1846	0.1256	0.148	0.0786	0.0121	0.0067
TOP	Precip	5.34	2.49	2.54	1.74	3.38	5.61	4.32	3.43	3.78	4.22	6.36	5.58
	*T*_max_	−5.90	−3.10	2.80	7.70	12.50	16.00	18.60	18.60	14.60	7.10	−0.50	−4.60
	*T*_min_	−13.30	−13.50	−8.20	−3.10	1.10	4.60	7.00	6.80	2.80	−1.10	−6.10	−11.00
	CDI	0.0321	0.0393	0.0683	0.0438	0.0941	0.188	0.1764	0.1732	0.1809	0.1666	0.0803	0.0422
LET	Precip	1.63	1.79	2.21	3.02	5.38	7.80	6.85	5.86	3.86	2.34	2.03	1.57
	*T*_max_	−0.70	−0.40	5.50	11.20	16.30	19.90	21.80	21.40	18.10	12.10	3.00	−0.80
	*T*_min_	−11.90	−12.60	−6.50	−1.40	3.10	7.30	8.80	7.80	3.90	−1.30	−7.40	−11.60
	CDI	0.0441	0.0405	0.0633	0.0861	0.1854	0.307	*0.2853*	*0.3087*	*0.1788*	*0.1432*	0.0821	0.0476
VAN	Precip	27.20	16.86	18.12	13.91	11.75	11.95	7.15	7.22	6.14	18.62	31.07	26.02
	*T*_max_	1.80	2.60	5.10	8.40	12.40	15.40	18.50	19.20	16.50	9.80	3.80	1.30
	*T*_min_	−2.80	−3.20	−1.30	1.60	4.20	7.00	9.90	10.00	7.30	3.60	−0.40	−2.70
	CDI	0.1139	0.1154	0.1475	0.2058	0.25	0.3764	0.391	0.3617	0.2831	0.2459	0.144	0.1101
ROC	Precip	10.85	9.27	7.60	8.35	8.18	9.90	8.44	10.66	10.04	11.26	10.90	9.74
	*T*_max_	−6.50	−8.40	−2.90	3.00	8.90	14.90	18.70	18.70	14.10	8.20	1.90	−3.40
	*T*_min_	−14.90	−17.00	−11.70	−3.60	0.50	5.10	8.90	10.30	5.70	1.40	−4.10	−9.80
	CDI	0.0221	0.0125	0.0452	0.1133	0.174	0.3148	0.3839	0.5064	0.3432	0.2024	0.1051	0.0502
ONE	Precip	1.53	1.58	2.33	2.37	3.84	7.16	5.41	3.16	3.40	1.62	1.88	1.68
	*T*_max_	−4.90	−3.10	4.60	11.30	17.30	22.40	24.50	24.50	19.20	11.30	1.10	−4.00
	*T*_min_	−15.50	−15.50	−7.40	−1.50	4.10	9.00	10.30	9.50	4.00	−3.10	−10.30	−15.40
	CDI	0.0269	0.0242	0.0627	0.0661	0.1236	0.3062	*0.254*	*0.1412*	*0.127*	*0.0807*	0.0589	0.0248
XIS	Precip	1.56	2.90	2.60	6.59	14.84	18.07	25.44	26.50	16.57	9.29	4.73	3.11
	*T*_max_	21.40	23.70	26.00	28.90	28.50	27.40	26.40	26.90	26.90	25.00	22.50	20.30
	*T*_min_	9.80	10.10	12.00	15.70	18.70	21.00	20.40	20.10	19.10	17.00	13.90	9.90
	CDI	0.1066	0.1932	0.1456	0.3517	0.7411	0.8761	0.8652	0.8607	*0.8512*	*0.7282*	*0.399*	0.2113
BOG	Precip	4.56	9.16	11.62	13.87	11.56	5.81	6.23	6.31	13.49	13.13	14.30	7.43
	*T*_max_	19.60	19.30	19.30	19.20	19.60	18.80	18.30	18.40	18.90	18.30	18.50	18.80
	*T*_min_	9.60	10.10	10.50	11.20	12.20	11.20	10.70	10.40	10.50	10.10	10.30	9.60
	CDI	0.261	0.4723	0.4294	0.5169	0.5202	0.3416	0.3739	0.3617	0.5269	0.4776	0.5048	0.3588
BIO	Precip	3.11	3.33	4.58	4.77	6.50	8.97	8.34	7.65	4.87	3.09	4.45	5.13
	*T*_max_	0.80	1.70	6.40	12.10	17.30	19.20	22.40	22.20	17.10	12.10	3.80	0.70
	*T*_min_	−3.60	−3.50	−0.60	2.30	7.10	10.00	12.40	12.00	8.50	3.70	−0.20	−2.50
	CDI	0.1024	0.1127	0.151	0.1733	0.2555	0.4353	0.4473	0.5156	0.3649	0.2313	0.1474	0.1079
SUM	Precip	3.11	3.33	4.58	4.77	6.50	8.97	8.34	7.65	4.87	3.09	4.45	5.13
	*T*_max_	0.80	1.70	6.40	12.10	17.30	19.20	22.40	22.20	17.10	12.10	3.80	0.70
	*T*_min_	−3.60	−3.50	−0.60	2.30	7.10	10.00	12.40	12.00	8.50	3.70	−0.20	−2.50
	CDI	0.1024	0.1127	0.151	0.1733	0.2555	0.4353	0.4473	0.5156	0.3649	0.2313	0.1474	0.1079
KOM	Precip	2.28	2.89	3.40	3.96	6.28	7.51	5.31	5.72	5.03	3.27	4.27	4.09
	*T*_max_	1.20	3.10	7.90	14.70	18.80	21.60	25.30	24.70	19.10	13.90	5.10	1.40
	*T*_min_	−2.80	−2.30	1.10	4.50	9.60	12.20	15.10	14.30	10.90	5.30	1.10	−1.80
	CDI	0.0987	0.1266	0.1519	0.1572	0.3186	0.4368	0.2992	0.4443	0.3772	0.2573	0.1698	0.1166
PAL	Precip	2.28	2.89	3.40	3.96	6.28	7.51	5.31	5.72	5.03	3.27	4.27	4.09
	*T*_max_	1.20	3.10	7.90	14.70	18.80	21.60	25.30	24.70	19.10	13.90	5.10	1.40
	*T*_min_	−2.80	−2.30	1.10	4.50	9.60	12.20	15.10	14.30	10.90	5.30	1.10	−1.80
	CDI	0.0987	0.1266	0.1519	0.1572	0.3186	0.4368	0.2992	0.4443	0.3772	0.2573	0.1698	0.1166
GIE	Precip	5.41	3.94	6.02	4.70	5.28	7.61	7.54	5.95	6.05	4.15	5.09	6.59
	*T*_max_	1.90	2.20	6.80	11.20	16.60	18.20	21.40	21.10	15.80	11.40	5.60	2.40
	*T*_min_	−1.30	−2.20	0.60	3.20	7.60	10.40	13.40	12.30	9.00	5.60	1.40	−0.60
	CDI	0.1262	0.1258	0.1681	0.1857	0.2253	0.4134	0.4676	0.4392	0.4065	0.2731	0.1776	0.1329
JEN	Precip	3.86	3.80	4.58	4.18	4.44	7.44	7.23	5.37	4.78	2.92	4.74	5.29
	*T*_max_	1.70	2.30	7.30	12.10	17.70	19.60	22.50	22.10	17.20	12.20	5.20	2.20
	*T*_min_	−2.10	−2.70	0.10	3.30	7.30	10.20	13.10	12.50	9.00	5.00	0.80	−1.00
	CDI	0.1194	0.123	0.1489	0.1677	0.2148	0.3764	0.4405	0.3962	0.3823	0.2524	0.1683	0.1283
KEN	Precip	5.86	11.23	14.43	27.27	21.95	15.29	11.67	17.35	15.51	20.92	13.21	10.89
	*T*_max_	28.40	28.90	29.10	27.70	26.40	26.10	26.00	26.70	28.00	28.20	28.20	28.10
	*T*_min_	12.80	13.10	14.10	14.50	14.40	14.10	13.60	13.30	13.00	14.20	14.20	12.90
	CDI	0.3105	0.5301	0.6646	*0.7941*	*0.7647*	*0.7106*	0.6566	0.7255	0.6663	0.7021	0.7107	0.4515
MAL	Precip	7.29	9.96	11.03	12.12	30.39	23.80	23.18	32.12	27.61	29.60	30.51	23.10
	*T*_max_	25.70	25.50	26.60	27.20	27.20	26.30	26.00	26.00	26.20	26.80	26.40	26.30
	*T*_min_	19.50	18.50	19.00	19.20	19.40	19.10	19.20	18.80	19.20	19.20	19.00	19.70
	CDI	*0.6476*	*0.5711*	*0.6202*	0.6266	0.8543	0.8334	0.8419	0.8357	0.8452	0.8543	0.8453	0.7291
NAM	Precip	0.00	0.00	0.00	0.12	0.00	0.00	0.00	0.00	0.00	0.00	0.00	0.00
	*T*_max_	19.60	22.90	27.80	32.90	37.00	40.10	39.20	38.30	36.60	32.00	26.10	20.40
	*T*_min_	5.60	8.30	12.00	16.90	20.80	23.50	23.60	23.70	21.40	16.80	11.10	7.20
	CDI	0.0144	0.0185	0.0242	0.0304	0.0317	0.033	0.0328	0.0327	0.0318	0.0287	0.0226	0.016
KUR	Precip	4.38	3.28	3.81	5.47	5.90	8.82	6.24	5.91	7.03	5.10	4.75	4.48
	*T*_max_	−4.40	−4.10	1.50	10.90	18.10	22.20	22.60	22.30	15.90	8.30	−0.80	−4.60
	*T*_min_	−9.60	−9.30	−4.30	3.10	8.30	12.80	14.20	12.70	8.30	2.30	−4.60	−8.80
	CDI	0.0515	0.0533	0.1045	0.2302	0.274	0.5028	0.4013	0.4099	0.3469	0.1913	0.0887	0.0496
SEN	Precip	0.05	0.05	0.01	0.03	0.07	0.83	3.77	9.68	10.05	1.51	0.00	0.06
	*T*_max_	30.55	32.30	34.60	35.75	37.20	37.05	34.20	33.55	33.85	35.65	33.75	30.90
	*T*_min_	14.05	16.40	17.90	19.05	20.70	22.45	23.40	24.35	24.15	23.15	19.55	16.10
	CDI	0.0277	0.0295	0.0302	0.0312	0.0325	0.0481	0.2005	0.6514	*0.6796*	*0.0878*	*0.0304*	0.0291
TAI	Precip	12.43	16.25	21.71	16.03	22.45	24.85	14.98	22.23	25.08	11.91	12.14	8.15
	*T*_max_	17.50	16.90	19.20	22.70	25.20	27.90	29.70	29.30	27.60	24.40	21.50	19.50
	*T*_min_	12.30	11.50	13.40	16.70	19.60	22.10	23.70	23.50	21.80	18.80	16.10	13.70
	CDI	0.5385	0.5123	0.6039	0.7267	0.8193	0.8577	0.8653	*0.9358*	*0.8976*	*0.7332*	0.6855	0.5995
CAR	Precip	7.67	4.77	6.75	4.05	4.33	6.74	7.80	8.62	8.75	8.11	7.44	7.53
	*T*_max_	5.70	5.20	9.30	11.70	16.70	18.00	21.40	21.00	16.90	13.50	8.90	6.20
	*T*_min_	1.70	0.40	2.70	4.10	7.90	10.80	13.80	12.80	10.50	7.70	4.50	2.80
	CDI	0.1827	0.1629	0.2007	0.1931	0.2128	0.3687	0.4696	0.4692	0.4874	0.3594	0.249	0.1972
SIL	Precip	9.09	6.01	5.35	5.97	4.15	4.72	4.89	4.88	6.27	8.44	6.38	7.96
	*T*_max_	6.50	6.10	9.30	11.60	15.90	18.50	21.00	20.80	17.20	13.20	9.00	6.60
	*T*_min_	1.50	0.70	2.50	3.60	6.70	9.30	12.00	11.20	8.80	6.20	3.20	1.60
	CDI	0.1869	0.1654	0.2032	0.2279	0.1708	0.2172	0.2865	0.2965	0.3861	0.3361	0.2328	0.1876
COL	Precip	0.85	1.33	3.58	3.54	6.30	6.23	4.20	4.06	3.38	2.31	1.88	0.88
	*T*_max_	4.90	5.90	10.80	15.40	19.80	25.90	28.60	27.70	23.30	17.10	9.30	4.30
	*T*_min_	−8.90	−7.70	−3.40	0.20	5.40	10.70	12.80	12.30	7.10	0.90	−4.70	−9.10
	CDI	0.032	0.0418	0.0804	0.0996	0.1988	0.2558	0.1512	0.1806	0.1857	0.1125	0.0918	0.0361
FLO	Precip	6.81	5.47	8.36	4.79	7.77	18.94	17.67	19.10	17.74	10.62	6.45	3.67
	*T*_max_	22.90	23.80	25.70	27.40	30.80	31.80	32.50	32.10	31.50	29.20	26.50	23.20
	*T*_min_	11.50	11.80	13.70	15.20	18.60	21.60	22.50	22.50	22.50	19.20	16.30	12.00
	CDI	0.4673	0.4399	0.4604	0.2806	0.4586	0.8612	*0.9186*	*0.9172*	*0.9443*	0.6969	0.5374	0.2893
